# Supplementary material for: Maturation of persistent and hyperpolarization-activated inward currents shapes the differential activation of motoneuron subtypes during postnatal development
Source: eLife. 2021 Nov 16;10:e71385. doi: 10.7554/eLife.71385 (PMC8641952; doi:10.7554/eLife.71385)
Supplement: Supplementary file 1. — Number of motoneurons (n) included in each subtype (Del: delayed; Imm: immediate) across postnatal weeks 1–3. Passive properties include whole-cell capacitance, tau, input resistance, and resting membrane potential (RMP). Recruitment properties: rheobase, derecruitment current, current firing range, voltage threshold of the first spike, and relative distance between RMP and spike threshold. Repetitive firing properties measured following recruitment on slow depolarizing current ramps include minimum firing rate, firing rate at 2× rheobase, maximum firing rate, and the slope of the sub-primary range (SPR). Single spike (action potential) properties including spike amplitude, rise time, and half width were measured from threshold, and medium afterhyperpolarization (mAHP) amplitude and half width, were elicited with a 10 ms square current pulse applied at 1.25× rheobase. Statistics: data were analysed using two-factor ANOVA with MN type (Del and Imm) and developmental week (weeks 1–3) as factors. Statistical analyses addressed three core questions: (1) Was there a difference between subtypes? (2) Did it change during development? (3) Do the subtypes mature differentially during development? Statistical results listing F and p values from two-factor ANOVA addressing these questions are listed sequentially: (1) main effect of subtype; (2) main effect of development; and (3) subtype × development interaction. All data are presented as mean ± SD (min, max). p-Values are derived from Holm–Sidak post hoc comparisons within weeks, between MN types. Superscript numbers denote significant differences from Holm–Sidak within MN types, between weeks (1: week 1; 2: week 2; 3: week 3). [file elife-71385-supp1.docx]

**Supplementary Table 1**

| **Postnatal Maturation of Fast and Slow Motoneuron Intrinsic Properties (257 cells from 95 animals)** | | | | | |
| --- | --- | --- | --- | --- | --- |
| **Parameter** | | **Week 1 (P1-4)^1^**  **57 cells, 18 animals** | **Week 2 (P7-12)^2^**  **122 cells, 42 animals** | **Week 3 (P14-20)^3^**  **81 cells, 35 animals** | **2W ANOVA**  **F, p** |
| Cells (n) | Del | 33 | 82 | 53 |  |
|  | Imm | 24 | 40 | 28 |  |
| **Passive Properties** | | | | | |
| Capacitance (pF) | Del | 285±94(112,542)^2,3^ | 494±211(139,1191)^1^ | 467±271(63,1220)^1^ | 32.1, 3.9e-8  9.9, 6.8e-5  3.6, 0.03 |
|  | Imm | 243±73(139,378) | 312±191(67,668) | 258±99(116,433) |  |
|  | P value | 0.9 | **3.6e-6** | **6.5e-6** |  |
| Tau (ms) | Del | 17.1±7.4(4.2,37.6)^3^ | 13.5±5.0(3.4,27.7) | 9.9±5.7(2.1,34.4)^1^ | 41.5,5.9e-10  1.1, 0.3  7.9, 4.7e-4 |
|  | Imm | 17.8±7.9(6.8,35.7)^2,3^ | 26.1±19.0(6.1,75.0)^1^ | 25.6±19.6(3.7,82)^1^ |  |
|  | P value | 0.97 | **1.8e-7** | **7.8e-8** |  |
| Passive Input Resistance (MΩ) | Del | 65.1±33.0(21.7,167.5) ^2,3^ | 30.8±15.9(10.4,114.1) ^1^ | 27.2±18.6(6.6,93.4)^1^ | 78.9, >1.0e-15  0.7, 0.5  10.6, 3.9e-5 |
|  | Imm | 76.6±35.5(30.4,114.1) ^3^ | 94.4±62.5(22.3,290.5) | 106.0±88.2(18.1,400) ^1^ |  |
|  | P value | 0.6 | **5.3x10e-12** | **1.2e-12** |  |
| Steady State Input Resistance (MΩ) | Del | 49.4±17.1(22.0,99.9) ^2,3^ | 27.2±18.4(2.8,99.4) ^1^ | 17.7±13.9(0.6,68) ^1^ | 52.9, 4.5e-12  3.7, 0.03  6.6, 0.002 |
|  | Imm | 57.2±18.4(17.2,90.0) | 73.5±65.4(17.8,326.0) | 59.9±42.5(15.7,180) |  |
|  | P value | 0.6 | **1.4e-10** | **6.1x10^-7** |  |
| Resting Membrane Potential (mV) | Del | -62.6±4.0(-69.0,-50.0) ^3^ | -63.9±4.2(-72.0,-52.0) ^3^ | -66.8±3.6(-77,-60.0) ^1,2^ | 25.5, 8.4e-7  10.4, 4.4e-5  4.4, 0.01 |
|  | Imm | -61.7±4.4(-69.0,-50.0) ^2^ | -58.3±5.4(-72.0,-52.0) ^1,3^ | -61.5±6.9(-73.0,-51.0) ^2^ |  |
|  | P value | 0.8 | **1.3e-8** | **3.5e-5** |  |
| **Recruitment Properties (Ramp)** | | | | | |
| Rheobase (pA) | Del | 349±214(118,1010) ^2,3^ | 550±254(149,1619) ^1,3^ | 816±631(127,2929) ^1,2^ | 45.4, 1.1e-10  3.4, 0.04  12.1, 9.6e-6 |
|  | Imm | 327±178(101,757) | 235±225(23,891) | 173±176(23,797) |  |
|  | P value | 0.8 | 1.0e-4 | 2.3e-12 |  |
| Derecruitment Current (pA) | Del | 1038±300(286,1501) ^2,3^ | 2372±752(967,4605) ^1,3^ | 3624±1374(1586,7828) ^1,2^ | 27.1, 4.0e-7  57.1, >1.0e-15  14.1, 1.7e-6 |
|  | Imm | 1290±344(413,1862) ^3^ | 1661±659(394,25430) | 2151±999(726,4023) ^1^ |  |
|  | P value | 0.99 | **0.001** | **1.7e-10** |  |
| Current Firing Range (pA) | Del | 937±297(210,1490) ^2,3^ | 1810±678(604,3776) ^1,3^ | 2853±1029(1136,6236) ^1,2^ | 24.7, 1.1e-6  70.1,>1.0e-15  3.4, 0.03 |
|  | Imm | 695±251(154,1089) ^2,3^ | 1396±571(311,2379) ^1,3^ | 1962±965(699,3904) ^1,2^ |  |
|  | P value | 0.99 | 0.08 | **7.0e-6** |  |
| First Spike TH (mV) | Del | -37.9±3.7(-44.8,-29.0) ^3^ | -38.8±3.3(-45.2,-31.2) ^3^ | -43.7±3.8(-51.8,-34.4) ^1,2^ | 7.4, 0.007  80.4, >1.0e-15  6.6, 0.002 |
|  | Imm | -36.6±3.1(-43.5,-30.2) ^2,3^ | -41.3±4.6(-51.2,-30.5) ^1,3^ | -47.2±4.5(-54.1,-35.7) ^1,2^ |  |
|  | P value | 0.4 | **0.02** | **5.3e-4** |  |
| Spike TH-RMP (mV) | Del | 24.5±5.4(11.3,22.9) | 24.2±4.8(10.2,23.8) | 26.5±5.3(17.6,25.9) | 35.6, 8.5e-9  5.7, 0.004  9.7, 9.2e-5 |
|  | Imm | 25.1±5.6(12.3,19.8) ^2,3^ | 17.9±7.0(3.8,30.3) ^1^ | 18.9±7.1(6.3,30.5) ^1^ |  |
|  | P value | 0.9 | **1.0e-8** | **6.0e-7** |  |
| **Repetitive Firing Properties on Ramp** | | | | | |
| Minimum Firing Rate (Hz) | Del | 10.9±3.7(6.9,24.0) ^3^ | 10.5±5.2(4.9,43.8) ^3^ | 6.9±2.2(4.5,15.2) ^1,2^ | 2.4, 0.1  15.1, 6.5e-7  6.7, 0.002 |
|  | Imm | 11.3±4.8(6.2,24.3) ^2,3^ | 7.1±2.4(2.4,13.4) ^1^ | 7.4±2.5(2.2,12.3) |  |
|  | P value | 0.99 | **0.01** | 0.99 |  |
| Firing Rate at 2x Rheobase (Hz) | Del | 24.7±5.8(14.2,38.6) | 28.9±8.3(13.1,53.2) ^3^ | 24.5±9.8(8.2,52.6) ^2^ | 33.7, 2.0e-8  1.9, 0.2  3.0, 0.05 |
|  | Imm | 21.2±5.3(13.3,38.5) | 17.9±9.6(3.4,45.3) | 17.1±12.8(5.4,61.4) |  |
|  | P value | 0.99 | **4.8e-8** | **0.009** |  |
| Maximum Firing Rate (Hz) | Del | 36.1±6.4(19.6,49.0) ^2,3^ | 45.6±9.4(27.5,83.3) ^1^ | 46.6±11.8(30.6,73.5) ^1^ | 7.3, 0.007  58.5, >1.0e-15  37.5, 7.0e-14 |
|  | Imm | 28.3±6.7(16.9,39.7) ^3^ | 37.6±10.6(20.9,60.2) ^3^ | 82.4±45.2(25.4,196) ^1,2^ |  |
|  | P value | 0.99 | 0.4 | 7.0e-15 |  |
| SPR FI slope (Hz/pA) | Del | 0.084±0.03(0.03,0.15) | 0.096±0.06(0.01,0.41) ^3^ | 0.07±0.05(0.02,0.24) ^2^ | 0.3, 0.6  2.5, 0.08  12.2, 8.7e-6 |
|  | Imm | 0.062±0.03(0.013,0.12) ^3^ | 0.065±0.02(0.03,0.11) ^3^ | 0.11±0.08(0.02,0.31) ^1,2^ |  |
|  | P value | 0.99 | **0.03** | **0.006** |  |
| **Single Spike Properties** | | | | | |
| Amplitude (mV) | Del | 75.5±4.3(66,84) | 73.0±5.3(60,87) | 74.8±6.8(61,89) | 6.5, 0.01  3.2, 0.04  2.0, 0.14 |
|  | Imm | 75.2±5.9(61,87) | 76.2±7.8(62,92) | 76.7±10.4(68,91) |  |
|  | P value | 0.99 | 0.1 | 0.2 |  |
| Spike rise time (ms) | Del | 0.77±0.15(0.40,1.0) ^2,3^ | 0.59±0.12(0.31,0.92) ^1,3^ | 0.47±0.08(0.34,0.65) ^1,2^ | 39.3, 1.6e-9  85.9, >1.0e-15  7.1, 0.001 |
|  | Imm | 0.98±0.28(0.40,1.49) ^2,3^ | 0.78±0.22(0.45,1.38) ^1,3^ | 0.50±0.18(0.28,1.2) ^1,2^ |  |
|  | P value | **2.6e-5** | **8.4x10e-8** | 0.8 |  |
| Spike Half width (ms) | Del | 0.99±0.22(0.53,1.39) ^2,3^ | 0.66±0.14(0.44,0.99) ^1,3^ | 0.57±0.11(0.38,0.82) ^1,2^ | 28.1, 2.5e-7  128, >1.0e-15  13.5, 2.7e-6 |
|  | Imm | 1.26±0.28(0.73,1.95) ^2,3^ | 0.89±0.34(0.48,2.38) ^1,2^ | 0.51±0.13(0.32,0.81) ^1,2^ |  |
|  | P value | **5.8e-6** | **3.2e-7** | 0.2 |  |
| AHP Amplitude (mV) | Del | 6.7±2.2(3.6,12.7) | 6.6±2.0(2.9,12.3) | 6.3±2.5(1.3,12.5) | 0.07, 0.78  6.4, 0.002  9.3, 1.3e-4 |
|  | Imm | 4.5. ±1.9(1.1,9.6) ^2,3^ | 7.3±2.5(2.5,11.9) ^1^ | 7.5±3.6(1.6,14.9) ^1^ |  |
|  | P value | **0.008** | 0.6 | 0.3 |  |
| AHP Half width (ms) | Del | 61.1±18.8(31.8,135.1) ^2,3^ | 43.3±12.7(22.1,80.1) ^1^ | 49.9±19.7(14.6,94.9) ^1^ | 38.8, 2.0e-9  1.1, 0.3  6.7, 0.001 |
|  | Imm | 66.9±20.2(24.7,107.4) | 75.8±31.3(32.1,152.3) | 67.7±32.6(24.7,136.2) |  |
|  | P value | 0.6 | **4.0e-11** | **6.1 e-3** |  |
