# Supplementary material for: Maturation of persistent and hyperpolarization-activated inward currents shapes the differential activation of motoneuron subtypes during postnatal development
Source: eLife. 2021 Nov 16;10:e71385. doi: 10.7554/eLife.71385 (PMC8641952; doi:10.7554/eLife.71385)
Supplement: Supplementary file 2. — Characteristics of the subthreshold membrane potential trajectory during slow depolarizing recruitment current ramps. Presented are the acceleration onset voltage, acceleration amplitude, passive recruitment current estimated based on the initial linear trajectory of the membrane potential, actual measured recruitment current, and an estimate of the underlying persistent inward current (ePIC) that produces the membrane potential acceleration. Delta I reflects the difference in derecruitment and recruitment currents on triangular depolarizing current ramps and provides an additional estimate of PIC. PICs were measured in voltage clamp during weeks 2–3 with the onset voltage, peak voltage, current amplitude, and densities measured. Sag conductance was estimated during weeks 1–3 and underlying h current (Ih) measured with amplitude and density at –70 mV and –110 mV presented. Time constant (tau) of Ih. Statistics: data were analysed using two-factor ANOVA with MN type (delayed and immediate) and developmental week (weeks 1–3) as factors. Statistical analyses addressed three core questions: (1) Was there a difference between subtypes? (2) Did it change during development? (3) Do the subtypes mature differentially during development? Statistical results listing F and p values from two-factor ANOVA addressing these questions are listed sequentially: (1) main effect of subtype; (2) main effect of development; (3) subtype × development interaction. All data are presented as mean ± SD (min, max). Number of cells (n) are included in brackets for each group. p-Values are derived from Holm–Sidak post hoc comparisons within weeks, between MN types. Superscript numbers denote significant differences from Holm–Sidak within MN types, between weeks (1: week 1; 2: week 2; 3: week 3). [file elife-71385-supp2.docx]

**Supplementary Table 2**

| **Postnatal Maturation of Active Properties in Fast and Slow Motoneurons** | | | | | |
| --- | --- | --- | --- | --- | --- |
| **Parameter** | | **Week 1 (P1-4)^1^** | **Week 2 (P7-12)^2^** | **Week 3 (P14-20)^3^** | **2W ANOVA**  **F, p** |
| **Subthreshold Membrane Potential Acceleration on Recruitment Ramp** | | | | | |
| Acceleration onset voltage (mV) | Del | -49.1±3.8(-53.9, -39.8) ^2,3^  (33) | -51.8±2.5(-57.2, -45.5) ^1^  (82) | -51.4±4.4(-59.1, -35.9) ^1^  (53) | 1.9, 0.2  24.9,1.4e-10  6.1, 0.003 |
|  | Imm | -48.0±4.4(-56.5, -38.2) ^2,3^  (24) | -52.0±3.4(-57.3,-44.2) ^1,3^  (40) | -55.6±3.1(-57.8, -47.0) ^1,2^  (28) |  |
|  | P value | 0.7 | 0.8 | **0.003** |  |
| Acceleration Amplitude (dV)  Spike TH – Acceleration onset (mV) | Del | 11.0±3.7(4.4,21.3)  (33) | 12.9±2.3(7.0,18.5)  (82) | 12.1±3.4(6.1,24.9)  (53) | 4.1, 0.04  0.9, 0.4  2.2, 0.1 |
|  | Imm | 11.6±4.2(3.8,20.6)  (24) | 11.0±4.1(3.8,22.2)  (40) | 10.5±3.4(3.4,17.6)  (28) |  |
|  | P value | 0.98 | 0.1 | 0.5 |  |
| Passive Rheobase (pA) | Del | 497±280(168,1267) ^2,3^  (33) | 1017±492(268,2560) ^1,3^  (82) | 1498±1100(272,4154) ^1,2^  (53) | 66.7, 1.7e-13  7.1, 0.001  15.8, 3.4e-7 |
|  | Imm | 433±218(115,985)  (24) | 411±392(57,1518)  (40) | 250±164(60,672)  (40) |  |
|  | P value | 0.9 | **6.8e-7** | **1.0e-15** |  |
| Actual Rheobase (pA) | Del | 349±214(118,1010) ^2,3^  (33) | 550±254(149,1619) ^1,3^  (82) | 816±631(127,2929) ^1,2^  (53) | 45.4, 1.1e-10  3.4, 0.04  12.1, 9.6e-6 |
|  | Imm | 327±178(101,757)  (24) | 235±225(23,891)  (40) | 173±176(23,797)  (28) |  |
|  | P value | 0.8 | **1.0e-4** | **2.3e-12** |  |
| ePIC  Passive-Actual Rheobase (pA) | Del | 150±107(-18,439) ^2,3^  (33) | 500±348(101,1855) ^1,3^  (82) | 727±579(137,3072) ^1,2^  (53) | 44.4, 1.8e-9  10.9, 3.0e-5  10.2, 5.6e-5 |
|  | Imm | 116±105(-32,389)  (24) | 174±216(5.0,1110)  (40) | 112±110(23,519)  (28) |  |
|  | P value | 0.99 | **4.9e-5** | **6.4e-14** |  |
| **Recruitment-derecruitment Hysteresis and Persistent Inward Current** | | | | | |
| Delta I Hysteresis (pA) | Del | -112±165(-510,123) ^2,3^  (16) | -280±129(-601, -10) ^1,3^  (56) | -339±181(-976, -71)  (53) | 85.9, <1.0e-15  5.8, 0.004  6.1, 0.003 |
|  | Imm | 7.7±110(-178,184)  (9) | -20±51(-147,37)  (21) | 9.2±99.3(309, -224)  (28) |  |
|  | P value | 0.2 | **7.1e-10** | **1.0e-16** |  |
| PIC onset (mV) | Del |  | -50.2±4.1(-59.7, -39.9)  (25) | -49.0±4.6(-59.0, -42.5)  (14) | 57.6, 2.2e-10  0.3, 0.6  0.2, 0.7 |
|  | Imm |  | -58.3±4.4(-67.0, -51.5)  (14) | -58.2±4.9(-64.1, -49.1)  (13) |  |
|  | P value |  | **5.0e-6** | **6.0e-6** |  |
| PIC peak (mV) | Del |  | -32.8±5.0 (-45.7, -22.7)  (25) | -30.2±6.3(-37.6, -19.4)  (14) | 38.6, 5.1e-8  0.85, 0.4  1.3, 0.3 |
|  | Imm |  | -39.6±5.4(-53.3, -32.0)  (14) | -39.9±4.7(-53.1, -34.3)  (13) |  |
|  | P value |  | **0.0032** | **0.0005** |  |
| PIC amplitude (pA) | Del |  | 386±236(60, 1055)  (24) | 502±205(175,950)  (14) | 17.9, 7.8e-5  5.8, 0.02  0.02, 0.9 |
|  | Imm |  | 164±121 (52,366)  (14) | 271±80(49,680)  (13) |  |
|  | P value |  | **0.007** | **0.03** |  |
| PIC density (pA/pF) | Del |  | 0.87±0.45(0.17,1.95) ^3^  (25) | 1.45±0.98(0.39,3.8) ^2^  (14) | 5.3, 0.02  13.8, 4.4e-4  0.3, 0.6 |
|  | Imm |  | 0.60±0.35 (0.1, 1.3)^3^  (14) | 1.08±0.47(0.23,1.9) ^2^  (13) |  |
|  | P value |  | 0.3 | 0.2 |  |
| **Sag and Ih** | | | | | |
| eSag Conductance (nS) | Del | 3.6±3.5(-5.5,12.9) ^3^  (33) | 6.5±13.1(-41.6,43.2)^3^  (82) | 31.9±35.9(-25.2,129.1) ^1,2^  (53) | 7.5, 0.007  7.3, 8.3e-4  5.2, 0.006 |
|  | Imm | 4.3±5.3(-2.2,25.2)  (24) | 6.0±15.8(-28.7,49.0)  (40) | 7.2±6.7(-2.6,28.7)  (28) |  |
|  | P value | 0.99 | 0.99 | **8.9e-7** |  |
| Ih amplitude  (-70 mV) (pA) | Del |  | -25±42(4.2, -113) ^3^  (15) | -119±103(-7.3, -275) ^2^  (19) | 14.4, 3.0e-4  5.3, 0.02  13.4, 5.3e-4 |
|  | Imm |  | -23±93(50.6, -116)  (12) | -1.9±14.4(23.1, -26.2)  (19) |  |
|  | P value |  | 0.98 | **2.0e-5** |  |
| Ih Density  (-70 mV) (pA/pF) | Del |  | -0.05±0.1(0.2, -0.2) ^3^  (15) | -0.53±0.85(-0.01, -3.0) ^2^  (19) | 3.6, 0.06  2.5, 0.1  6.0, 0.02 |
|  | Imm |  | -0.12±0.1(0.15, -0.4)  (12) | -0.015±0.07(0.1, -0.1)  (19) |  |
|  | P value |  | 0.99 | **0.01** |  |
| Ih amplitude  (-110 mV) (pA) | Del |  | -776±535(-67, -1472) ^3^  (15) | -1176±557(-131, -2749) ^2^  (18) | 29.5, 1.1e-6  1.3, 0.3  6.1, 0.02 |
|  | Imm |  | -449±330(-51, -902)  (12) | -302±196(-94, -640)  (19) |  |
|  | P value |  | 0.1 | **3.0e-7** |  |
| Ih Density  (-110 mV) (pA/pF) | Del |  | -1.8±1.4(-0.25, -5.5) ^3^  (15) | -4.6±5.8(-0.5, -21.4) ^2^  (18) | 4.9, 0.03  2.0, 0.2  4.0, 0.05 |
|  | Imm |  | -1.6±1.0(-0.4, -3.4)  (12) | -1.2±0.76(-0.17, -2.6)  (19) |  |
|  | P value |  | 0.99 | **0.015** |  |
| Ih tau  (-110 mV) (s) | Del |  | 0.37±0.11(0.3,0.7) ^3^  (15) | 0.22±0.07(0.1,0.3) ^2^  (19) | 50.1, 4.1e9  31.2, 1.0e-6  50.1, 4.1e-9 |
|  | Imm |  | 0.51±0.09(0.4,0.7) ^3^  (12) | 0.41±0.05(0.3,0.5) ^2^  (19) |  |
|  | P value |  | **0.005** | **1.5e-9** |  |
