# Supplementary material for: Maturation of persistent and hyperpolarization-activated inward currents shapes the differential activation of motoneuron subtypes during postnatal development
Source: eLife. 2021 Nov 16;10:e71385. doi: 10.7554/eLife.71385 (PMC8641952; doi:10.7554/eLife.71385)
Supplement: Supplementary file 3. — Comparison of intrinsic properties measured in studies on motoneuron subtypes (current work; Leroy et al., 2014) and those that pooled all lumbar motoneurons studied (Quinlan et al., 2011; Nakanishi and Whelan, 2010; Smith and Brownstone, 2020). Data are presented as mean ± SD; Nakanishi and Whelan, 2010 are presented as mean ± SEM. [file elife-71385-supp3.docx]

**Supplementary Table 3**

|  |  | **Week 1** | | | | **Week 2** | | | | | **Week 3** | |
| --- | --- | --- | --- | --- | --- | --- | --- | --- | --- | --- | --- | --- |
| **Property** | MN Type | Sharples and Miles, 2021 | Smith and Brownstone  2020 | Quinlan et al., 2011 | Nakanishi and Whelan, 2010 | Sharples and Miles, 2021 | Smith and Brownstone  2020 | Leroy et al., 2014 | Quinlan et al., 2011 | Nakanishi and Whelan, 2010 | Sharples and Miles, 2021 | Smith and Brownstone  2020 |
| Age range (P) |  | 1-4 | 2-3 | 0-5 | 0-3 | 7-14 | 6-7 | 6-10 | 6-12 | 8-11 | 14-20 | 14-21 |
| N’s | Del | 31 | 17 | 16 | 72 | 82 | 30 | 63 | 20 | 36 | 53 | 18 |
|  | Imm | 23 |  |  |  | 40 |  | 31 |  |  | 28 |  |
| Capacitance  (pF) | Del | 285±94 | 223±71 | 273±81 | 88±5.3 | 494±211 | 310±140 |  | 321±83 | 99±8.3 | 467±271 | 405±217 |
|  | Imm | 243±73 |  |  |  | 312±191 |  |  |  |  | 258±99 |  |
| Input Res.  (MΩ) | Del | 65.1±33 | 79±47 | 62±17 | 100±6 | 30.8±15.9 | 52±32 |  |  | 87±10 | 27.2±18.6 | 31±32 |
|  | Imm | 76.6±35 |  |  |  | 94.4±62.5 |  |  |  |  | 106.0±88.2 |  |
| RMP (mV) | Del | -62.6±4.0 | -65±4.4 | -57±4 |  | -63.9±4.2 | -65±3.2 | -64±3 | -60±6 |  | -66.8±3.6 | -67±2.7 |
|  | Imm | -61.7±4.4 |  |  |  | -58.3±5.4 |  | -65 ±3 |  |  | -61.5±6.9 |  |
| Rheobase (pA) | Del | 349±214 | 450±380 | 368±175 | 157±8.4 | 550±254 | 520±270 | 1100±600 | 374±18 | 288±40 | 816±631 | 1500±1100 |
|  | Imm | 327±178 |  |  |  | 235±225 |  | 600±500 |  |  | 173±176 |  |
| Spike TH (mV) | Del | -37.9±3.7 | -36±9.5 | -29±5 | -52±8.4 | -38.8±3.3 | -37±7.2 | -33±7 | -30±7 | -56±2.1 | -43.7±3.8 | -35±8.2 |
|  | Imm | -36.6±3.1 |  |  |  | -41.3±4.6 |  | -44±7 |  |  | -47.2±4.5 |  |
| Spike Amplitude (mV) | Del | 75.5±4.3 | 69±7.8 |  | 74±1.2 | 73.0±5.3 | 68±38 | 89±13 |  | 75±2.5 | 74.8±6.8 | 70±8.5 |
|  | Imm | 75.2±5.9 |  |  |  | 76.2±7.8 |  | 84±11 |  |  | 76.7±10.4 |  |
| Spike HW/*dur. (ms) | Del | 0.99±0.22 | 1.2±0.2 | 1.5±0.6* | 1.33±0.05 | 0.66±0.14 | 1.0±0.2 | 1.4±0.5* | 1.1±0.3* | 0.97±0.07 | 0.57±0.11 | 0.8±0.2 |
|  | Imm | 1.26±0.28 |  |  |  | 0.89±0.34 |  | 1.7±0.4* |  |  | 0.51±0.13 |  |
| Spike Rise Time (ms) | Del | 0.77±0.15 |  |  | 0.98±0.04 | 0.59±0.12 |  |  |  | 0.81±0.06 | 0.47±0.08 |  |
|  | Imm | 0.98±0.28 |  |  |  | 0.78±0.22 |  |  |  |  | 0.50±0.18 |  |
| mAHP Amplitude (mV) | Del | 6.7±2.2 | 5.3±2.4 | 9.4±2.3 |  | 6.6±2.0 | 4.1±1.9 |  | 9.1±3 |  | 6.3±2.5 | 3.0±1.6 |
|  | Imm | 4.5. ±1.9 |  |  |  | 7.3±2.5 |  |  |  |  | 7.5±3.6 |  |
| mAHP Half width (ms) | Del | 61.1±18.8 | 67±22 |  | 45±11 | 43.3±12.7 | 74±39 | 27±9 |  | 49±20 | 49.9±19.7 | 74±31 |
|  | Imm | 66.9±20.2 |  |  |  | 75.8±31.3 |  | 42±9 |  |  | 67.7±32.6 |  |
| Min. FR (Hz) | Del | 10.9±3.7 |  |  |  | 10.5±5.2 |  |  |  |  | 6.9±2.2 |  |
|  | Imm | 11.3±4.8 |  |  |  | 7.1±2.4 |  |  |  |  | 7.4±2.5 |  |
| Max FR (Hz) | Del | 36.1±6.4 | 44±8 |  |  | 45.6±9.4 | 46±19 |  |  |  | 46.6±11.8 | 56±17 |
|  | Imm | 28.3±6.7 |  |  |  | 37.6±10.6 |  |  |  |  | 82.4±45.2 |  |
| ePIC ramp  Delta I (pA) | Del | -112±165 |  | -52±134 |  | -280±129 |  |  | -117±195 |  | -339±181 |  |
|  | Imm | 7.7±110 |  |  |  | -20±51 |  |  |  |  | 9.2±99.3 |  |
| PIC Onset Voltage (mV) | Del |  |  | -46±5 |  | -50.2±4.1 |  |  | -42±10 |  | -49.0±4.6 |  |
|  | Imm |  |  |  |  | -58.3±4.4 |  |  |  |  | -58.2±4.9 |  |
| PIC Amplitude (pA) | Del |  |  | 149±96 |  | 386±236 |  |  | 214±127 |  | 502±205 |  |
|  | Imm |  |  |  |  | 164±121 |  |  |  |  | 271±80 |  |
| PIC Density (pA/pF) | Del |  |  | 0.52±0.27 |  | 0.87±0.45 |  |  | 0.7±0.4 |  | 1.45±0.98 |  |
|  | Imm |  |  |  |  | 0.60±0.35 |  |  |  |  | 1.08±0.47 |  |
